# Usefulness of intraoperative ultrasound examination for laparoscopic right-side colon cancer surgery: a propensity score-matched study

**DOI:** 10.1038/s41598-023-49867-8

**Published:** 2023-12-17

**Authors:** Hiroaki Kasashima, Tatsunari Fukuoka, Gen Tsujio, Atsushi Sugimoto, Ken Yonemitsu, Kisyu Kitayama, Yasuhito Iseki, Masatsune Shibutani, Kiyoshi Maeda

**Affiliations:** Department of Gastroenterological Surgery, Osaka Metropolitan University Graduate School of Medicine, 1–4–3 Asahi-machi Abeno-ku, Osaka, Osaka 545-8585 Japan

**Keywords:** Cancer, Medical research

## Abstract

Complete mesocolic excision (CME) with central vascular ligation (CVL) in laparoscopic surgery for right-sided colon cancer (RSCC) requires a precise understanding of the vascular anatomy. The efficacy of intraoperative ultrasound (IUS) in the identification of blood vessels for RSCC surgery was not evaluated. The aim of this study was to compare the intraoperative and short-term outcomes of CME with CVL with or without IUS by laparoscopic surgery for RSCC. We performed IUS on 26 patients of RSCC and compared with a total of 124 patients who underwent the surgery for RSCC at our institution. Propensity score matching (PSM) was performed to reduce the confounding effects to imbalances in the use of IUS. The IUS identified the main feeding artery and the accompanying vein in all 26 cases. After PSM, the amount of intraoperative blood loss in the IUS group was significantly lower than that in the conventional group (5 ml vs. 30 ml, p = 0.035) and no significant difference of the postoperative complications was observed. The IUS reduced the risk of bleeding in the surgery for RSCC. The IUS is a safe and feasible technique that help the surgeons for anatomical understandings under real-time condition in the laparoscopic surgery of RSCC.

## Introduction

Complete mesocolic excision (CME), in conjunction with central vascular ligation (CVL), which emphasizes the importance of sharp dissection along the mesocolic plane with central ligation of the main blood vessels at their roots, was first described by Hohenberger et al.^1^. Although CME with CVL in laparoscopic surgery is now an essential technique for colon cancer surgery^2–4^, CVL is the most difficult part of the procedure, especially for the right sided colon cancer surgery, owing to the complex vascular anatomy^5^. Additionally, to complete the adequate lymph node dissection for right sided colon cancer (RSCC), a precise understanding of the anatomy of the main vessels, including the superior mesenteric artery/vein (SMA/SMV), the root of the middle colonic artery (MCA), and the ileocolic artery/vein (ICA/ICV) is needed^6,7^. The efficacy of intraoperative ultrasound (IUS) in the identification of blood vessels for RSCC surgery was reported by Sadakari et al.^8^. In 2021; however, no studies to date have compared conventional surgery for RSCC to those with IUS.

The aim of this study was to compare the intraoperative and short-term outcomes of a propensity-matched cohort undergoing CME with CVL with or without IUS by laparoscopic surgery for RSCC.

## Results

The patient characteristics and operative outcomes are summarized in Table 1. IUS was performed in 26 cases (the IUS group) for RSCC and the main feeding artery and the accompanying vein were identifiable in all cases, while conventional techniques were used in 98 cases (the conventional group). Differences in patient characteristics and tumor factors between the two groups before PSM were observed according to sex before PSM. After PSM, with patient characteristics and tumor factors (location and depth) included as covariates, 25 patients were included in this study. Importantly, the amount of intraoperative blood loss in the IUS group was significantly lower than that in the conventional group (5 ml vs. 30 ml, p = 0.037) in the entire cohort, and similar results were observed in the matched cohort. In fact, 4 of the 13 cases with heavy bleeding (more than 100 ml) in the conventional group were due to vascular injury around the SMV, while no case of bleeding more than 100 ml was observed in the IUS group.

**Table 1:** Patients’ characteristics, clinicopathological factors and operative outcomes.

Factors	Entire cohort			Matched cohort		
	The conventional group (n = 98)	The IUS group (n = 26)	*p*-value	The conventional group (n = 25)	The IUS group (n = 25)	*p*-value
Patients’ characteristics and clinicopathological factors						
Age (years)						
< 75	57	17	0.65	16	16	< 0.999
≤ 75	41	9		9	9	
Sex, n						
Male	75	13	0.014	14	13	< 0.999
Female	23	13		11	12	
Body mass index (kg/m^2^)						
Median (range)	22.7 (14.8–36.5)	23.0 (17.1–35.6)	0.89	21.7 (17.7–32.5)	22.0 (17.1–35.6)	0.85
History of laparotomy						
Yes	31	9		6	8	
No	67	17	0.81	19	17	0.75
Tumor location, n						
Cecum	20	3	0.23	2	3	< 0.999
Ascending colon	56	13		14	13	
Transverse colon	22	10		9	9	
Tumor depth, n						
T1,2	48	9	0.27	8	9	< 0.999
T3,4	50	17		17	16	
Histology						
Pap, tub1, tub2	92	25	< 0.999	24	24	< 0.999
Por, muc	6	1		1	1	
Operative outcomes						
Number of dissected lymph nodes						
Median (range)	18 (1–100)	22 (7–72)	0.34	17 (1–45)	21 (7–55)	0.267
Operative duration (min)						
Median (range)	235 (142–365)	287 (145–458)	< 0.01	240 (158–365)	287 (145–458)	0.056
Intraoperative blood loss (ml)						
Median (range)	30 (5–350)	5 (5–95)	0.037	30 (5–200)	5 (5–95)	0.035
Transfusion						
Yes	1	0	< 0.999	0	0	< 0.999
No	97	26		25	25	

The operation time in the IUS group was longer than that in the conventional group (287 min vs. 235 min, p = 0.0004) before propensity score matching; however, after PSM, it tended to be longer, but the difference was not statistically significant. The analysis for the association between surgical skill (blood loss and operative time) and the level of the surgeon using endoscopic surgical skill qualification system clarified that there was not significant difference regardless of whether the qualified surgeon performed the surgery or not (Fig. S1a–d).

There was no significant difference in the the number of dissected lymph nodes between the IUS and conventional groups (21.0 [7–55] versus 17.0 [1–45], p = 0.27) after PSM.

The postoperative complications are summarized in Table S1. No statistically significant difference was observed between the two groups in anastomotic leakage, re-operation, or postoperative bleeding.

## Discussion

IUS is mainly used in liver surgery to identify the tumor location and distance from the main vessels^9^ and it is also applied for the detection of liver metastases in colorectal surgery^10^. Sadakari et al. previously reported the technical pitfall of IUS for colorectal surgery to identify the mesenteric artery^8,11^; however, its usefulness was not evaluated by a statistical analysis. This is the first report to analyze the effectiveness of IUS for RSCC.

The main advantages of IUS for colorectal surgery are the prevention of injury and bleeding of the SMV and its tributaries during CVL for RSCC. The National Clinical Database of Japan has reported that the operative mortality rate of surgery for RSCC is unexpectedly high (2.2%), mainly due to accidental bleeding from the SMV^12^. Furthermore, laparoscopic CME with CVL for RSCC increased the risk of bleeding from the SMA and SMV^13^. It was also reported that injury to the SMV was more frequent in patients who received CVL than in those who did not receive CVL^14^. We hypothesized that IUS would enable us to visualize the location of the main vessels and their tributaries, not only the SMA/SMV and ICA/ICV, but also the RCA/MCA or jejunal vein which sometimes run above the SMA^15^. Furthermore, IUS can be performed under actual intra-operative conditions. This is a significant advantage, especially in patients with adhesion or abundant visceral fat. Although a 3D-CT is also helpful for obtaining an anatomical understanding of key vessels, the vascular anatomy would be changed by the surgical procedure during CME and traction by the assistant’s hands. A combination of 3D-CT and IUS can be applied complementarily to achieve safe CVL and accurate dissection of the regional lymph nodes. In particular, identification of the MCA root using IUS enables surgeons to complete appropriate lymph node dissection for RSCC in safety. Lastly, IUS can be performed if preoperative 3D-CT angiography is unavailable because of allergy, renal dysfunction, or asthma. In addition to being a complementary method for the preoperative evaluation of the vascular anatomy in surgery for RSCC, IUS can be employed an alternative method.

The present study was associated with some limitations. Firstly, this was a single-center study. The efficacy of IUS should be investigated in multicenter, prospective randomized trials in future studies. Second, the current sample size was too small to investigate for the surgical outcomes, including morbidity and mortality. Importantly, however, this propensity score-matched study proved that IUS—a non-invasive intraoperative evaluation that is easy to perform- reduced the risk of bleeding in the surgery for RSCC. Therefore, despite these limitations, we believe that in suitable cases managed by a skilled surgical team, IUS should be performed in CME with CVL for RSCC.

## Materials and methods

From January 2018 to May 2023, 124 patients diagnosed with pathologically proven RSCC underwent laparoscopic surgery at Osaka Metropolitan University Hospital were enrolled in this study. Surgical procedures depended on tumor location and main feeding artery were determined according to the concept described in the Japanese Society for Cancer of the Colon and Rectum guidelines 2019^16^. The surgeries for RSCC without IOUS was classified “the conventional group” and those with IOUS was “the IUS group”. IUS was conducted in continuous 26 patients when IUS probe was available. This study protocol was reviewed and approved by the Ethical Committee of Osaka Metropolitan University Graduate School of Medicine (approval number 926).

The surgical procedure for RSCC was divided into four steps, as previously reported^8^. (1) the cranial approach (release of the bursa omentalis, dissection and mobilization of the lateral attachments and hepatic flexure); (2) the retroperitoneal approach (mobilization of the cecum and ascending colon from the retroperitoneum); (3) the medial approach (dissection of the main vessels and regional lymph nodes); and (4) specimen extraction and anastomosis. In step (3), IUS was performed after conditioning the surgical view with assistance.

In this study, a laparoscopic ultrasound probe (ARIETTA70^®^ and L43K^®^; HITACHI ALOKA Medical, Japan) was inserted via a 12-mm port into the left-upper abdomen. The SMA/SMV and ICA/ICV in the mesentery were scanned from the root of the transverse mesocolon and marked using gentian violet (1st scanning), followed by dissection of the mesocolon at the peripheral side of the branching ICV, and the SMV surface was exposed. The ICA was then clipped and cut at the root from the anterior ICA to the SMV and at the right border of the SMV from the posterior ICA to the SMV. For right hemicolectomy, the MCA was also scanned, and its root was identified from the SMA. After a 2nd scan and marking, the mesocolon was dissected to the cranial side above the SMA, and the #213 and #223LNs were dissected at the root of the RCA and MCA with ligation of the RCA and the right branch of the MCA. It would be better to use gentian violet in the place to be resected, as it was referred to as carcinogens.

**Figure 1:**
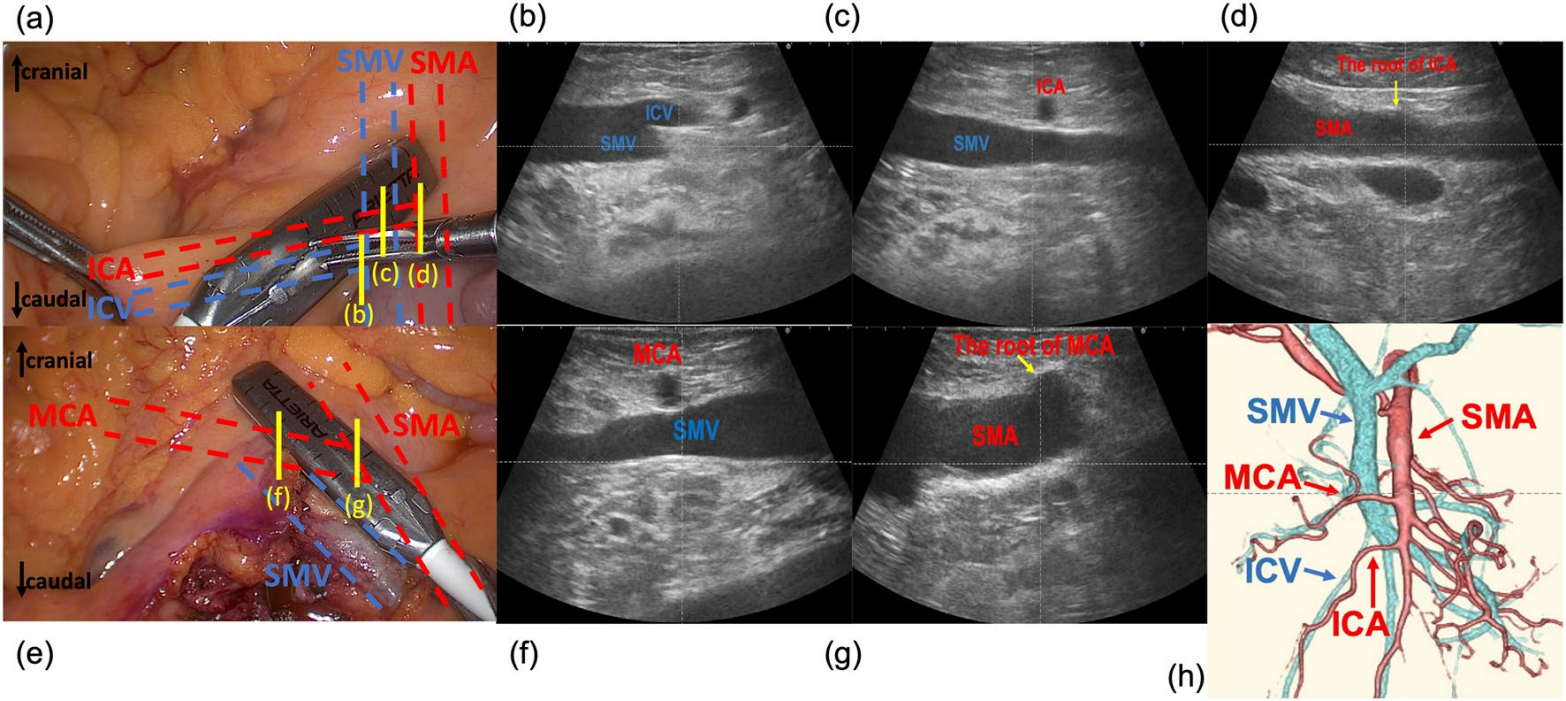
Procedures of intraoperative ultrasound examination for RSCC. (**a**–**d**) 1st scan: (**a**) the intraoperative image of 1st scan. The SMA and SMV, which pulled lateral side by the assistance, were scanned from their periphery toward the roots. (**b**) at the root of the SMV where ICV was branched. (**c**) above the SMV crossed by the ICA. (**d**) at the root of the SMA where ICA was branched. (**e–g**) 2nd scan: (**e**) the intraoperative image of 2nd scan. The transverse mesocolon was pulled cranial side by the assistance, and MCA was scanned from its periphery toward the root. (**f**) Above the SMV crossed by the MCA. (**g**) At the root of the SMA where MCA was branched. (**h**) 3D-CT angiography showed the ICA and MCA anterior to the SMV. SMA superior mesenteric artery, SMV superior mesenteric vein, ICV ileocolic vein, ICA ileocolic artery, MCA the middle colic artery, 3D-CT three- dimensional computed tomography.

Figure 1 shows representative images of IUS for the laparoscopic right hemicolectomy. IUS was performed during medial approach (Fig. 1a–d, 1st scan: Fig. 1e–g, 2nd scan: Fig. 1h, 3D-CT angiography).

The following variables were analyzed: age, sex, body mass index, history of laparotomy, tumor location, tumor depth, histology, number of dissected lymph nodes, duration of operation, intraoperative blood loss, transfusion, anastomotic leakage, re-operation, postoperative bleeding at the anastomotic site, and mortality. Intraoperative blood loss and duration of operation were statistically analyzed separately for Endoscopic Surgical Skill Qualification System-qualified surgeon and non-qualified surgeon in the conventional group and the IUS group, respectively. Endoscopic Surgical Skill Qualification System is administered by the Japanese Society of Endoscopic Surgery.

All patients without an unavailable condition (such as allergy or asthma) underwent a preoperative three-dimensional computed tomography (3D-CT) angiogram to assess and analyze 3D images of the SMA/SMV and their branches. Laparoscopic surgery for RSCC was performed on all patients using a standardized technique^17^ with five ports (12-mm umbilical port for the camera, 12-mm port for the surgeon’s right hand located at the left-upper abdomen and the remaining three 5-mm ports for the surgeon’s left hand and both the assistant’s hands (Fig. S2). If IUS is difficult due to the angle of US probe, small midline incision in advance, which will be needed for extracorporeal anastomosis, is optional for alternative 12-mm port next to the port for the camera through the same incision.

Written informed consent was obtained from all participants. This study was conducted in according with the principles of the Declaration of Helsinki. All statistical analyses were performed with EZR (Saitama Medical Center, Jichi Medical University, Saitama, Japan), which is a graphical user interface for R (The R Foundation for Statistical Computing, Vienna, Austria)^18^. *P* < 0.05 was considered to be statistically significant. To reduce the impact of selection bias and potential confounding, which is associated with non-randomized observational studies, we performed propensity score matching (PSM). The propensity scores were estimated using multivariate logistic regression models. The matched baseline information was as follows: age, sex, tumor location, tumor depth, histological tumor type. Patients were matched 1:1 by the neighbor matching method via a caliper width with a standard deviation of 0.2.

### Supplementary Information

Supplementary Figure S1.

Supplementary Figure S2.

Supplementary Video 1.

Supplementary Table S1.

Supplementary Legends.

## Data Availability

The full manuscript data has been read and approved by all authors and collaborators. Anonymized research data are available from the corresponding author upon reasonable request.
