# Supplementary figures and images for: Usefulness of intraoperative ultrasound examination for laparoscopic right-side colon cancer surgery: a propensity score-matched study
Source: Sci Rep. 2023 Dec 17;13:22440. doi: 10.1038/s41598-023-49867-8 (PMC10725876; doi:10.1038/s41598-023-49867-8)

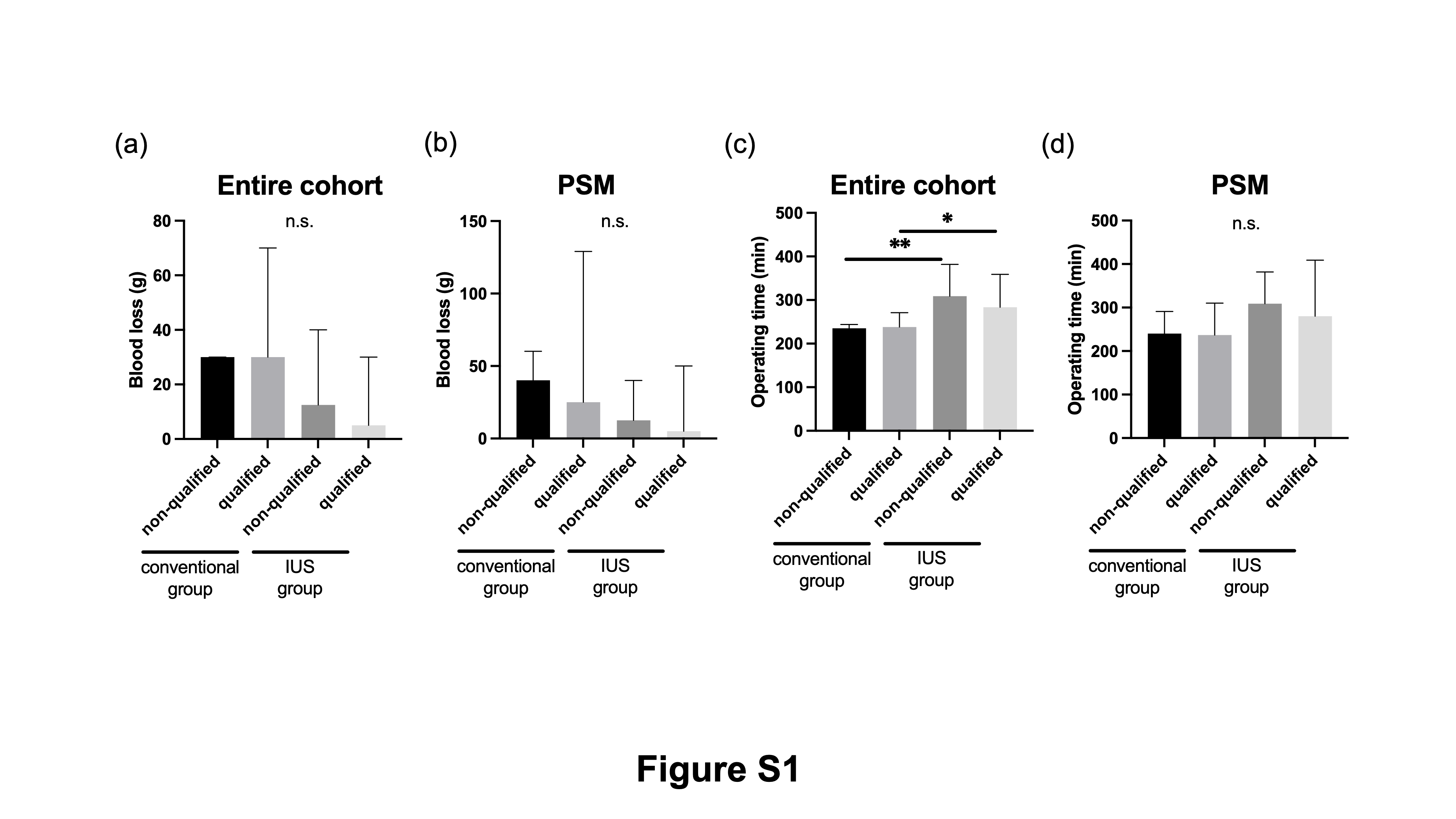

Supplement: Supplementary file 1 — Supplementary Figure S1. [file 41598_2023_49867_MOESM1_ESM.tiff]

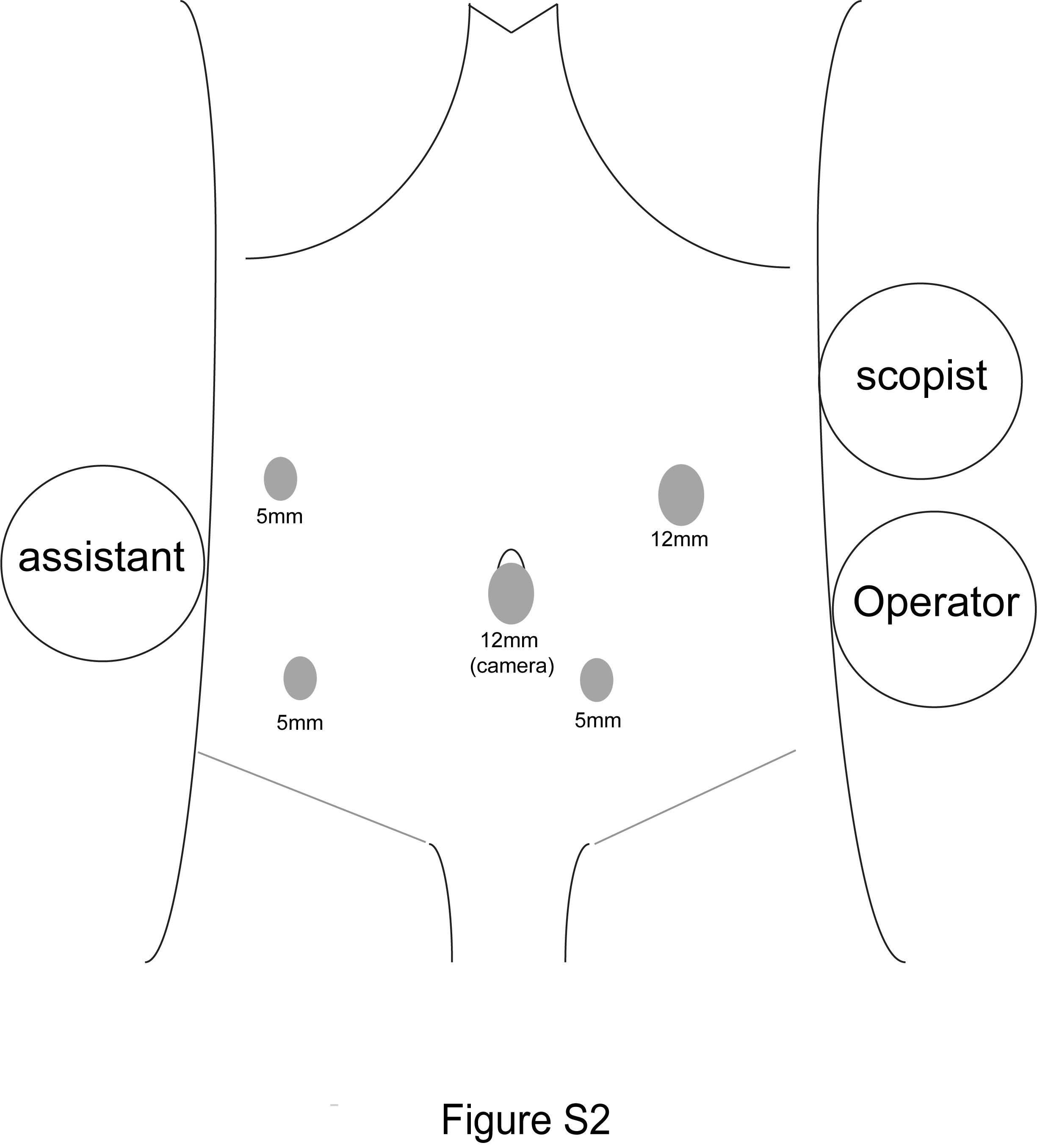

Supplement: Supplementary file 2 — Supplementary Figure S2. [file 41598_2023_49867_MOESM2_ESM.tif]
