# Supplementary material for: Usefulness of intraoperative ultrasound examination for laparoscopic right-side colon cancer surgery: a propensity score-matched study
Source: Sci Rep. 2023 Dec 17;13:22440. doi: 10.1038/s41598-023-49867-8 (PMC10725876; doi:10.1038/s41598-023-49867-8)
Supplement: Supplementary file 4 — Supplementary Table S1. [file 41598_2023_49867_MOESM4_ESM.docx]

**Table S1.** Postoperative complication

|  | Entire cohort | | | Matched cohort | | |
| --- | --- | --- | --- | --- | --- | --- |
| **Factors** | The conventional group (n=98) | The IUS group (n=26) | *p*-Value | The conventional group (n=25) | The IUS group (n=25) | *p*-Value |
| Anastomotic leakage | |  |  |  |  |  |
| n (%) | 3 (3.0%) | 0 (0%) | >0.99 | 1 (4.0%) | 0 (0%) | >0.99 |
| Re-operation |  |  |  |  |  |  |
| n (%) | 0 (0%) | 0 (0%) | N/A | 0 (0%) | 0 (0%) | N/A |
| Postoperative bleeding | |  |  |  |  |  |
| n (%) | 3 (3.0%) | 0 (0%) | >0.99 | 1 (4.0%) | 0 (0%) | >0.99 |
| Mortality |  |  |  |  |  |  |
| n (%) | 0 (0%) | 0 (0%) | N/A | 0 (0%) | 0 (0%) | N/A |
