# Supplementary material for: Usefulness of intraoperative ultrasound examination for laparoscopic right-side colon cancer surgery: a propensity score-matched study
Source: Sci Rep. 2023 Dec 17;13:22440. doi: 10.1038/s41598-023-49867-8 (PMC10725876; doi:10.1038/s41598-023-49867-8)
Supplement: Supplementary file 5 — Supplementary Legends. [file 41598_2023_49867_MOESM5_ESM.docx]

**Fig.S1** **Association between surgical skill (blood loss and operative time) and the level of the surgeon**

(a,b) Intraoperative blood loss in entire cohort (a), and matched cohort (b). (c,d) Operative time in entire cohort (c), and matched cohort (d). PSM Propensity score matching.

**Fig.S2** **Placement of surgical port for the laparoscopic surgery of right-sided colon cancer**

A 12-mm camera port was placed in the umbilical incision. The other 12-mm port was placed in the left lateral abdomen and three 5-mm ports were placed in the right-upper, right-lower, and left-lower abdominal regions. The operator was usually on the left and the assistant was on the right side of the patient, and the scopist was on the cranial side of the patient.
